# Spasmodic Contractions with Hypertrophy of the Retractor Muscles of the Penis of a Bull

**Published:** 1897-12

**Authors:** D. S. White

**Affiliations:** College of Veterinary Medicine, Ohio State University


### SPASMODIC CONTRACTIONS WITH HYPERTROPHY OF THE RETRACTOR MUSCLES OF THE PENIS OF A BULL.

By D. S. White, D.V.M.,
COLLEGE OF VETERINARY MEDICINE, OHIO STATE UNIVERSITY.

In April, 1896, a full-blooded, fifteen months' old Jersey bull was brought to the free clinic of the University with the anamnesis that it had never been able to serve a cow. The owner of the animal maintained that the penis, instead of being protruded from the prepuce during the act of coitus, was misdirected into a pouch formed by a fold of the skin of the lower abdominal wall. A careful examination of the animal, however, failed to divulge any such sac, nor at that time could anything else be found to explain the patient's inability to fully protrude the penis during an erection. The owner was advised, therefore, to take the animal home and inform us when one of his cows should come in heat, so that an examination could be made while the bull was in the act of covering. A few days afterward we went out to the farm and witnessed the animal attempt the act of copulation. The bull seemed to possess unusual sexual desire for one of his breed. At the beginning of a leap, in covering the cow, the penis was protruded about two inches beyond the orifice of the prepuce; but as an entrance was about to be made into the vulva it was drawn back again into the sheath and the ejaculation performed therein. The retraction seemed to be effected forcibly. An examination of the retractor muscles of the penis was then made; at their attachment to the S-shaped curve in the corpus cavernosum these muscles could be plainly felt, and, as the sexual excitement became greater, they seemed to be thrown into tonic spasmodic contractions, drawing the penis far back into the sheath. A diagnosis of "spasms of the retractor penis muscles" was made, and as the condition, which appeared to be permanent, would render the bull worthless for breeding purposes, it was suggested that the muscles be severed by operation at the S-curve. After overcoming the owner's objections, he fearing a prolapsus penis would result, the animal was cast and the muscles severed at a point about one inch from their attachment to the posterior curve. On cutting through it was noted that the muscles were hypertrophic, forming two distinctly marked semi-conical bands of about double the normal size. The muscular fibres were plainly seen on cross section as pale bundles of muscular tissue; the ligamentous structure correspondingly not so well developed as was found in the specimens from other animals of the bull kind examined by us afterward.

The operation, which proved to be very simple, being done with aseptic precautions, the wound healed per prima. The bull was used for service some seventeen days later, when a slight temporary prolapsus penis occurred, but which soon disappeared. The bull has since stood at the head of a herd of nearly forty cows, and has since the operation begotten a number of calves.

In the literature available to me I can find no record of another case of this kind, nor have we in our experience seen it duplicated.

